# Coding complete genomes of an iridovirus and two parvoviruses identified in lab-reared social spiders (*Stegodyphus dumicola*)

**DOI:** 10.1128/mra.00739-24

**Published:** 2024-10-14

**Authors:** Sydney Millerwise, Michael C. Lund, Kara Schimidlin, Simona Kraberger, Noa Pinter-Wollman, Arvind Varsani

**Affiliations:** 1 School of Life Sciences, Arizona State University, Tempe, Arizona, USA; 2 The Biodesign Center for Fundamental and Applied Microbiomics, Arizona State University, Tempe, Arizona, USA; 3 Department of Ecology and Evolutionary Biology, University of California, Los Angeles, California, USA; 4 Center for Evolution and Medicine, Arizona State University, Tempe, Arizona, USA; 5 Structural Biology Research Unit, Department of Integrative Biomedical Sciences, University of Cape Town, Rondebosch, Cape Town, South Africa; Portland State University, Portland, Oregon, USA

**Keywords:** social spiders, iridovirus, parvoviruses, house crickets

## Abstract

Coding complete genomes of an iridovirus (194,403 nts) and two parvoviruses (4,689, 3,764 nts) were identified in social spiders (*Stegodyphus dumicola*). The iridovirus and one of the parvovirus are most closely related to those from house crickets (*Acheta domesticus*), whereas the other is most closely related to one from a social spider.

## ANNOUNCEMENT

There is limited information on viruses associated with social spiders (*Stegodyphus dumicola*); thus, we undertook a pilot project to identify viruses in a lab population of these spiders. Spider colonies were collected in Kalkrand, Namibia, in March 2018 and shipped to UCLA (Permit # RPIV00632019) where they were maintained in the lab. The lab colonies were primarily fed house crickets (*Acheta domesticus*) from several local commercial sources. Deceased spiders (unknown cause) were shipped to the lab at ASU toward the end of July 2018. Whole bodies of 20 social spiders were homogenized with a mortar and pestle in sterile SM buffer (50 mM Tris–HCl, 10 mM MgSO4, 0.1 M NaCl, pH 7.5). The homogenate was sequentially filtered through 0.45- and 0.2-µm syringe filters. Viral DNA was extracted from 200 µL of the filtrate using the Roche high pure viral nucleic acid kit (Roche, USA) following the manufacturer’s instructions. The viral DNA was amplified using rolling circle amplification (RCA) with the TempliPhi amplification kit (GE Healthcare, USA). The RCA products were combined with the viral nucleic acid at a 1:1 ratio, and ~10 ng of DNA was used to prepare Illumina sequencing libraries using the Nextera DNA flex kit (Illumina, USA) and were sequenced on an Illumina NovaSeq 6000 (2 × 150 bp paired-end library), yielding 14,922,720 paired-end reads. Raw reads were quality-checked and trimmed using Trimmomatic v0.39 (1); these were *de novo* assembled using MEGAHIT v. 1.2.9 (2). The *de novo* assembled contigs (>1,000 nts) were analyzed using DIAMOND BLASTx (3) against a viral RefSeq protein database (version 220). We identified three contigs that correspond to invertebrate associated viruses (families *Iridoviridae* and *Parvoviridae*) (Table 1). The genomes were annotated using Cenote-Taker 3 (4), and the annotations were manually checked with reference sequences in Geneious Prime 2024.0.5 (Dotmatics, USA). All genome-wide pairwise identities were determined using SDT v1.2 (5). Default parameters were used for all bioinformatics tools. The genomes were determined to be coding complete based on the reference genomes available in GenBank. Of the total reads, ~950,000 reads map to the three genomes identified in this study (Table 1), and these are deposited in SRA accession (SRR29083913).

**TABLE 1:** Summary of query coverage, reads mapped, and top NCBI BLASTn hit for the iridovirus and two parvoviruses identified in this study

						Top blast hit			
Family	Genus	Accession #	Genome coverage	# of mapped reads	GC content	Accession #	E-value	Query coverage	% identity
*Iridoviridae*	*Iridovirus*	PP847201	165.7	220,057	27.70%	OK181107	0	100%	99.69%
*Parvoviridae*	*Ambidensovirus*	PP847202	13,311.40	475,620	37.30%	KF275669	0	99%	99.67%
	unclassified	PP847203	9,400.20	244,060	47.30%	BK066809	0	100%	95.74%

**Fig 1:**
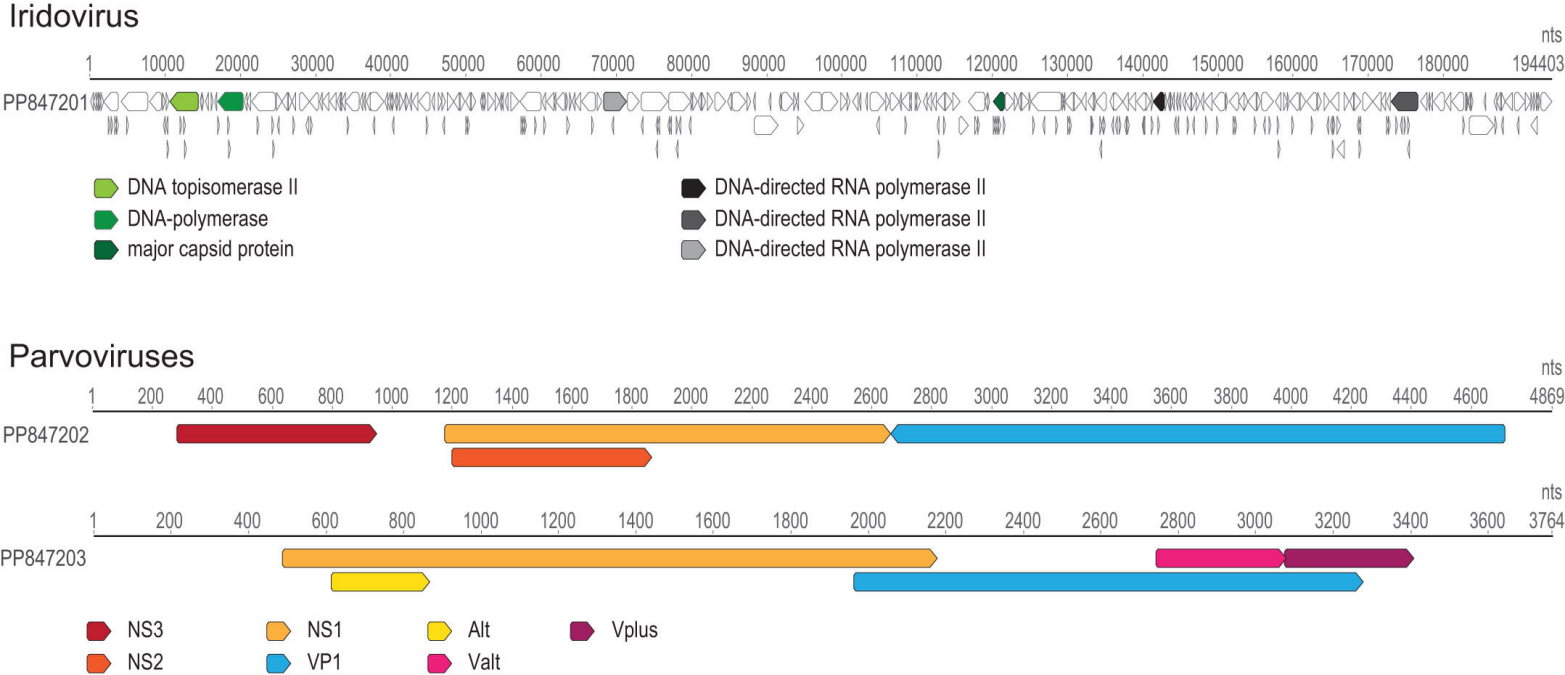
Genome organization of the iridovirus and the two parvoviruses. Some of the core ORFs and their encoded proteins are highlighted.

Iridoviruses have linear, double-stranded DNA viruses (103–220 kbp) with terminal redundancies. They are classified in six genera across two subfamilies with members of *Alphairidovirinae* infecting ectothermic vertebrates and *Betavirinae* infecting invertebrates. Although some iridovirus infections can cause blue or green iridescence on the exterior of invertebrates, most infections do not result in this phenotype (6, 7). The iridovirus genome (Fig. 1; accession # PP847201) we identified shares 98.43%–99.69% pairwise identity with two iridoviruses from house crickets (MN081869 and OK181107) (7, 8) and shares 75.15%–76.49% pairwise identity with invertebrate iridescent virus 6 genomes (AF303741 and MT862761) (9, 10).

Parvoviruses are single-stranded linear DNA viruses with genomes of ~4–6 kb (11). The two parvoviruses (Fig. 1), PP847202 and PP847203, shared 95.74% and 99.67% from *Acheta domesticus* mini ambidensovirus (KF275669) from house cricket (12) and parvovirus stegodyphus3780 (BK066809) from social spiders (13), respectively.

In summary, we detected predator–prey interactions between social spiders and the crickets they consumed by identifying two viruses most closely related to other cricket-associated viruses. We also identified a virus that is very similar (sharing 95.74% identity) to one identified from 15 bodies of social spiders (13).

## Data Availability

The sequences identified in this study have been deposited in GenBank under accession numbers # PP847201, PP847202, and PP847203. Raw reads can be found in SRA project # PRJNA1112455, BioSample # SAMN41427585 with SRA accession # SRR29083913.
